# Supplementary material for: Metaproteomics of saliva identifies human protein markers specific for individuals with periodontitis and dental caries compared to orally healthy controls
Source: PeerJ. 2016 Sep 14;4:e2433. doi: 10.7717/peerj.2433 (PMC5028799; doi:10.7717/peerj.2433)
Supplement: Supplemental Information 1 — List of bacterial genera and species identified. [file peerj-04-2433-s001.docx]

Bacterial genera distribution

| TaxName | Frequency | | | Abundance | | |
| --- | --- | --- | --- | --- | --- | --- |
|  | Caries | Healthy | Periodontitis | Caries | Healthy | Periodontitis |
| Streptococcus | 10 | 10 | 10 | 20.48 | 19.22 | 16.49 |
| Prevotella | 10 | 10 | 10 | 18.20 | 18.56 | 18.79 |
| Veillonella | 10 | 10 | 10 | 16.93 | 13.36 | 12.78 |
| Rothia | 10 | 10 | 10 | 10.20 | 11.21 | 8.73 |
| Neisseria | 10 | 10 | 10 | 9.38 | 9.57 | 9.17 |
| Actinomyces | 10 | 10 | 10 | 4.53 | 3.55 | 5.32 |
| Haemophilus | 10 | 10 | 10 | 2.52 | 5.71 | 3.54 |
| Leptotrichia | 10 | 10 | 10 | 1.74 | 1.75 | 4.80 |
| Lactobacillus | 10 | 10 | 10 | 1.66 | 1.12 | 0.41 |
| Fusobacterium | 10 | 10 | 10 | 1.26 | 1.47 | 3.78 |
| Porphyromonas | 10 | 10 | 10 | 0.91 | 1.29 | 1.70 |
| Selenomonas | 10 | 10 | 10 | 0.61 | 1.00 | 1.67 |
| Gemella | 10 | 10 | 10 | 0.54 | 0.79 | 0.77 |
| Mycoplasma | 10 | 10 | 10 | 0.42 | 0.45 | 0.29 |
| Oribacterium | 10 | 10 | 10 | 0.31 | 0.25 | 0.22 |
| Stomatobaculum | 10 | 10 | 10 | 0.30 | 0.21 | 0.32 |
| Alloprevotella | 9 | 10 | 10 | 0.29 | 0.53 | 0.35 |
| Megasphaera | 10 | 9 | 9 | 0.20 | 0.11 | 0.11 |
| Corynebacterium | 10 | 9 | 10 | 0.17 | 0.13 | 0.49 |
| Granulicatella | 10 | 10 | 10 | 0.09 | 0.11 | 0.06 |
| Atopobium | 9 | 10 | 9 | 0.07 | 0.04 | 0.06 |
| Tannerella | 7 | 4 | 9 | 0.05 | 0.01 | 0.13 |
| Capnocytophaga | 5 | 3 | 6 | 0.03 | 0.02 | 0.03 |
| Treponema | 3 | 3 | 8 | 0.03 | 0.00 | 0.08 |
| Campylobacter | 8 | 9 | 8 | 0.01 | 0.01 | 0.01 |
| Abiotrophia | 8 | 8 | 7 | 0.01 | 0.02 | 0.01 |
| Lachnoanaerobaculum | 7 | 6 | 6 | 0.01 | 0.02 | 0.11 |
| Bacteroides | 2 | 6 | 3 | 0.00 | 0.00 | 0.01 |
| Parvimonas | 0 | 2 | 5 | 0.00 | 0.01 | 0.03 |

Bacterial species distribution

| TaxName | Frequency | | | Abundance | | |
| --- | --- | --- | --- | --- | --- | --- |
|  | Caries | Healthy | Periodontitis | Caries | Healthy | Periodontitis |
| Prevotella histicola | 10 | 10 | 10 | 4.47 | 3.59 | 3.17 |
| Rothia mucilaginosa | 10 | 10 | 10 | 3.67 | 4.31 | 2.91 |
| Veillonella atypica | 10 | 10 | 10 | 3.23 | 2.25 | 2.20 |
| Prevotella melaninogenica | 10 | 10 | 10 | 2.29 | 2.82 | 3.18 |
| Streptococcus salivarius | 10 | 10 | 10 | 2.05 | 1.16 | 0.92 |
| Veillonella dispar | 10 | 10 | 10 | 1.95 | 1.51 | 1.52 |
| Lactobacillus salivarius | 10 | 10 | 10 | 1.66 | 1.12 | 0.41 |
| Streptococcus parasanguinis | 10 | 10 | 10 | 1.10 | 1.09 | 0.64 |
| Haemophilus parainfluenzae | 10 | 10 | 10 | 1.00 | 2.92 | 1.67 |
| Prevotella scopos | 10 | 10 | 10 | 0.86 | 0.91 | 0.73 |
| Veillonella sp. oral taxon 158 | 10 | 10 | 10 | 0.65 | 0.75 | 0.82 |
| Neisseria subflava | 7 | 8 | 9 | 0.58 | 0.63 | 0.48 |
| Actinomyces graevenitzii | 10 | 10 | 10 | 0.51 | 0.40 | 0.56 |
| Mycoplasma salivarium | 10 | 10 | 10 | 0.42 | 0.45 | 0.29 |
| Neisseria mucosa | 9 | 9 | 10 | 0.40 | 0.49 | 0.47 |
| Actinomyces sp. oral taxon 172 | 10 | 10 | 10 | 0.36 | 0.29 | 0.39 |
| Stomatobaculum longum | 10 | 10 | 10 | 0.30 | 0.21 | 0.32 |
| Porphyromonas sp. oral taxon 279 | 10 | 10 | 10 | 0.26 | 0.44 | 0.49 |
| Lachnospiraceae bacterium oral taxon 082 | 10 | 8 | 10 | 0.26 | 0.25 | 0.32 |
| Prevotella sp. oral taxon 473 | 9 | 10 | 10 | 0.23 | 0.41 | 0.29 |
| Leptotrichia buccalis | 10 | 10 | 10 | 0.23 | 0.25 | 0.56 |
| Streptococcus mitis | 10 | 10 | 10 | 0.22 | 0.34 | 0.16 |
| Leptotrichia wadei | 10 | 8 | 10 | 0.22 | 0.17 | 0.76 |
| Gemella sanguinis | 10 | 10 | 10 | 0.20 | 0.27 | 0.32 |
| Megasphaera micronuciformis | 10 | 9 | 9 | 0.20 | 0.11 | 0.11 |
| Fusobacterium periodonticum | 9 | 8 | 9 | 0.19 | 0.25 | 0.55 |
| Oribacterium sinus | 10 | 10 | 9 | 0.18 | 0.13 | 0.10 |
| Selenomonas sputigena | 8 | 8 | 9 | 0.18 | 0.17 | 0.42 |
| Corynebacterium matruchotii | 10 | 9 | 10 | 0.17 | 0.13 | 0.49 |
| Fusobacterium nucleatum | 10 | 10 | 10 | 0.15 | 0.14 | 0.42 |
| Prevotella pallens | 10 | 10 | 10 | 0.14 | 0.28 | 0.31 |
| candidate division TM7 single-cell isolate TM7a | 10 | 9 | 9 | 0.13 | 0.12 | 0.33 |
| Porphyromonas catoniae | 10 | 7 | 10 | 0.11 | 0.03 | 0.21 |
| Actinomyces odontolyticus | 9 | 10 | 10 | 0.11 | 0.07 | 0.07 |
| Streptococcus oligofermentans | 10 | 10 | 10 | 0.11 | 0.11 | 0.11 |
| Selenomonas flueggei | 8 | 8 | 8 | 0.11 | 0.20 | 0.27 |
| Selenomonas sp. oral taxon 149 | 10 | 10 | 9 | 0.10 | 0.19 | 0.20 |
| Streptococcus infantis | 10 | 9 | 10 | 0.09 | 0.18 | 0.12 |
| Atopobium parvulum | 9 | 10 | 9 | 0.07 | 0.04 | 0.06 |
| Streptococcus australis | 10 | 9 | 8 | 0.07 | 0.06 | 0.04 |
| Tannerella sp. oral taxon BU063 | 7 | 4 | 9 | 0.05 | 0.01 | 0.13 |
| Streptococcus cristatus | 4 | 8 | 5 | 0.05 | 0.03 | 0.01 |
| Granulicatella adiacens | 10 | 10 | 7 | 0.04 | 0.04 | 0.01 |
| Leptotrichia sp. oral taxon 215 | 8 | 7 | 10 | 0.04 | 0.06 | 0.13 |
| Prevotella sp. oral taxon 306 | 7 | 8 | 6 | 0.04 | 0.02 | 0.01 |
| Leptotrichia shahii | 7 | 7 | 8 | 0.03 | 0.04 | 0.18 |
| Streptococcus vestibularis | 5 | 3 | 5 | 0.03 | 0.01 | 0.01 |
| Prevotella veroralis | 8 | 7 | 9 | 0.02 | 0.04 | 0.02 |
| Prevotella saccharolytica | 5 | 8 | 8 | 0.02 | 0.06 | 0.03 |
| Prevotella oris | 6 | 5 | 7 | 0.02 | 0.01 | 0.01 |
| Prevotella oralis | 4 | 5 | 4 | 0.02 | 0.01 | 0.02 |
| Campylobacter concisus | 8 | 9 | 8 | 0.01 | 0.01 | 0.01 |
| Streptococcus gordonii | 4 | 6 | 5 | 0.01 | 0.01 | 0.01 |
| Gemella haemolysans | 3 | 6 | 4 | 0.01 | 0.01 | 0.01 |
| Porphyromonas gingivalis | 3 | 7 | 8 | 0.01 | 0.01 | 0.31 |
| Alloprevotella rava | 2 | 5 | 2 | 0.01 | 0.04 | 0.01 |
| Abiotrophia defectiva | 8 | 8 | 7 | 0.01 | 0.02 | 0.01 |
| Actinomyces sp. oral taxon 180 | 7 | 4 | 2 | 0.01 | 0.00 | 0.00 |
| Capnocytophaga sp. oral taxon 329 | 2 | 3 | 5 | 0.01 | 0.02 | 0.02 |
| Lachnoanaerobaculum saburreum | 7 | 6 | 6 | 0.01 | 0.02 | 0.11 |
| Prevotella micans | 4 | 5 | 7 | 0.01 | 0.01 | 0.03 |
| Fusobacterium necrophorum | 3 | 7 | 3 | 0.01 | 0.01 | 0.03 |
| Prevotella multisaccharivorax | 3 | 5 | 3 | 0.01 | 0.01 | 0.01 |
| Lachnospiraceae bacterium oral taxon 500 | 5 | 5 | 9 | 0.01 | 0.02 | 0.03 |
| Alloprevotella tannerae | 4 | 7 | 7 | 0.01 | 0.01 | 0.02 |
| Prevotella buccalis | 4 | 7 | 7 | 0.01 | 0.03 | 0.02 |
| Prevotella salivae | 7 | 7 | 7 | 0.01 | 0.01 | 0.01 |
| Neisseria flavescens | 1 | 4 | 2 | 0.01 | 0.01 | 0.00 |
| Prevotella nigrescens | 4 | 5 | 7 | 0.01 | 0.01 | 0.02 |
| Selenomonas sp. oral taxon 138 | 2 | 0 | 4 | 0.01 | 0.00 | 0.01 |
| Neisseria elongata | 2 | 1 | 2 | 0.01 | 0.00 | 0.01 |
| Bacteroides pyogenes | 2 | 6 | 3 | 0.00 | 0.00 | 0.01 |
| Prevotella baroniae | 3 | 3 | 8 | 0.00 | 0.00 | 0.01 |
| Leptotrichia goodfellowii | 6 | 6 | 5 | 0.00 | 0.00 | 0.00 |
| Granulicatella elegans | 4 | 7 | 6 | 0.00 | 0.01 | 0.01 |
| Prevotella intermedia | 3 | 1 | 1 | 0.00 | 0.00 | 0.00 |
| Leptotrichia sp. oral taxon 225 | 1 | 1 | 4 | 0.00 | 0.00 | 0.00 |
| Neisseria sp. oral taxon 014 | 2 | 0 | 4 | 0.00 | 0.00 | 0.00 |
| Actinomyces cardiffensis | 1 | 0 | 4 | 0.00 | 0.00 | 0.01 |
| Fusobacterium sp. oral taxon 370 | 0 | 2 | 8 | 0.00 | 0.00 | 0.04 |
| Treponema medium | 0 | 2 | 5 | 0.00 | 0.00 | 0.02 |
