# Supplementary figures and images for: Metaproteomics of saliva identifies human protein markers specific for individuals with periodontitis and dental caries compared to orally healthy controls
Source: PeerJ. 2016 Sep 14;4:e2433. doi: 10.7717/peerj.2433 (PMC5028799; doi:10.7717/peerj.2433)

**A**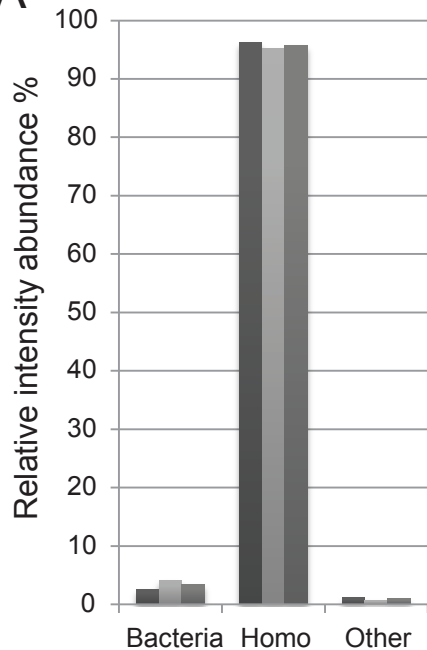**B**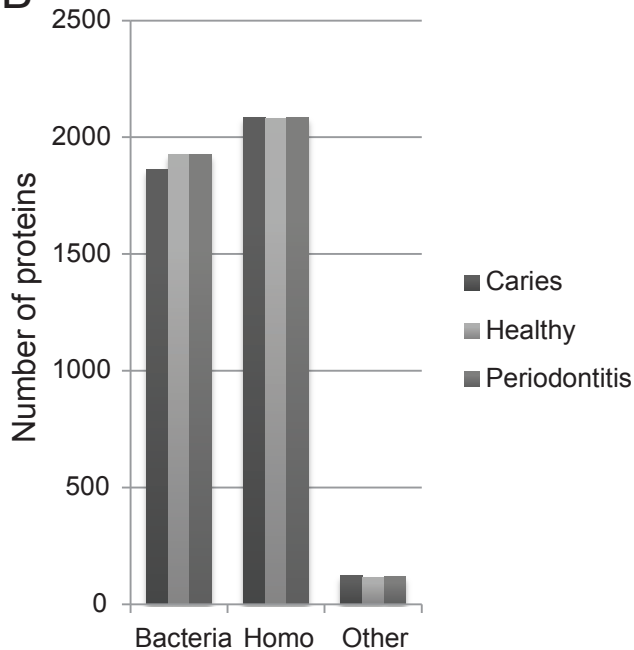

Supplement: Supplemental Information 2 — Intensity and protein abundance between groups shows no significant differences. [file peerj-04-2433-s002.pdf]

# KEGG pathway characterization

# associated protein-groups 0 1000 2000 3000

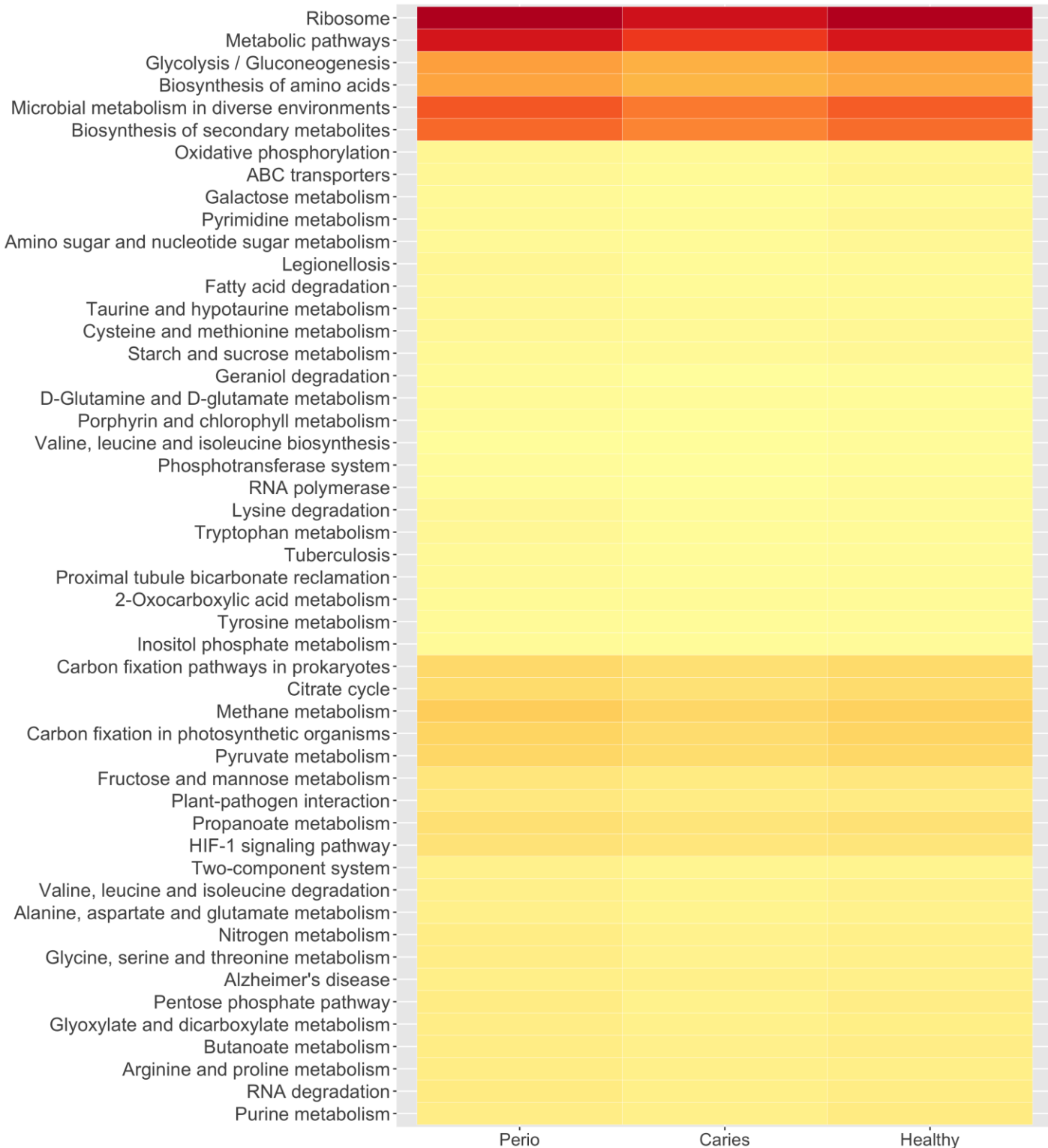

Supplement: Supplemental Information 3 — KEGG pathway enrichment for bacterial proteins shows no significant changes between groups. [file peerj-04-2433-s003.pdf]
